# Supplementary material for: IL-1 Generated by Oral Squamous Cell Carcinoma Stimulates Tumor-Induced and RANKL-Induced Osteoclastogenesis: A Possible Mechanism of Bone Resorption Induced by the Infiltration of Oral Squamous Cell Carcinoma
Source: Int J Mol Sci. 2022 Dec 30;24(1):688. doi: 10.3390/ijms24010688 (PMC9821332; doi:10.3390/ijms24010688)
Supplement: Supplementary file 1 [file ijms-24-00688-s001.zip › ijms-2058794-supplementary.pdf]

International Journal of *Molecular Sciences*
MDPI

Supplementary Materials

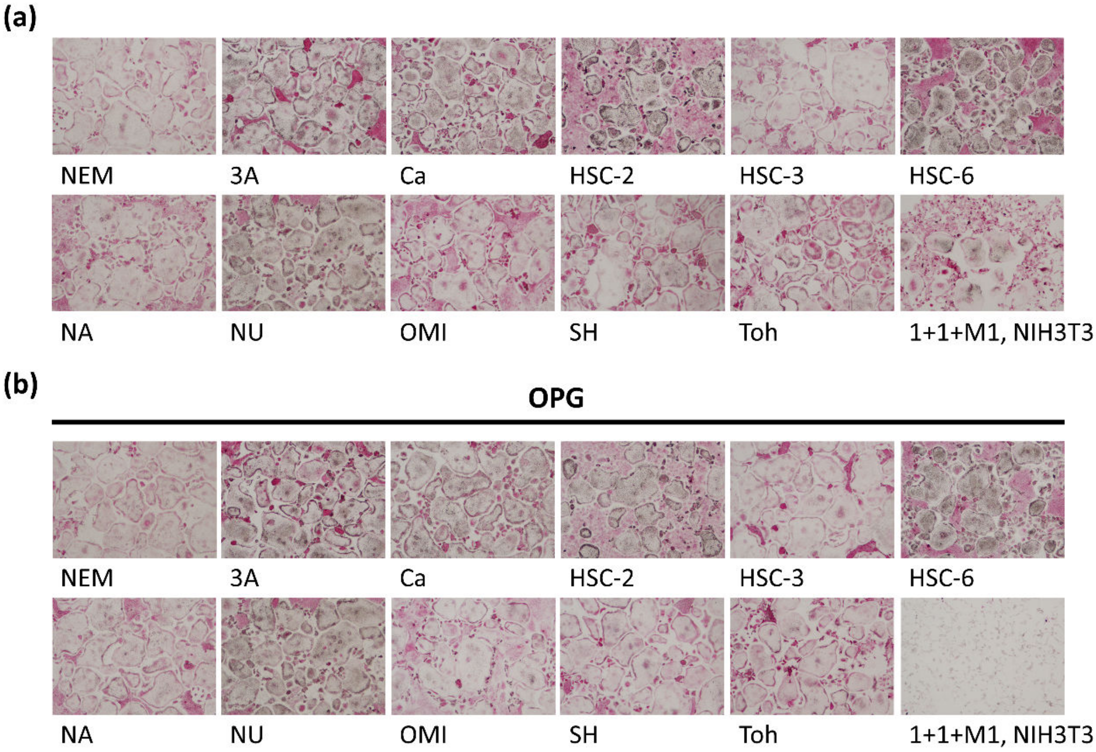

Figure S1. Representative views of TRAP staining 5 days after the coculture of various kinds of OSCC cells with OPCs. (a) OSCC cell lines, NEM, 3A, Ca, HSC-2, HSC-3, HSC-6, NA, NU, OMI, SH, or Toh were cocultured with OPCs. As a control, 1+1+M1, NIH3T3 cells overexpressing human RANKL and mouse M-CSF were also cocultured with OPCs. (b) For some of these cultures, OPG was further added to evaluate the effects of the RANKL inhibitor on osteoclastogenesis.

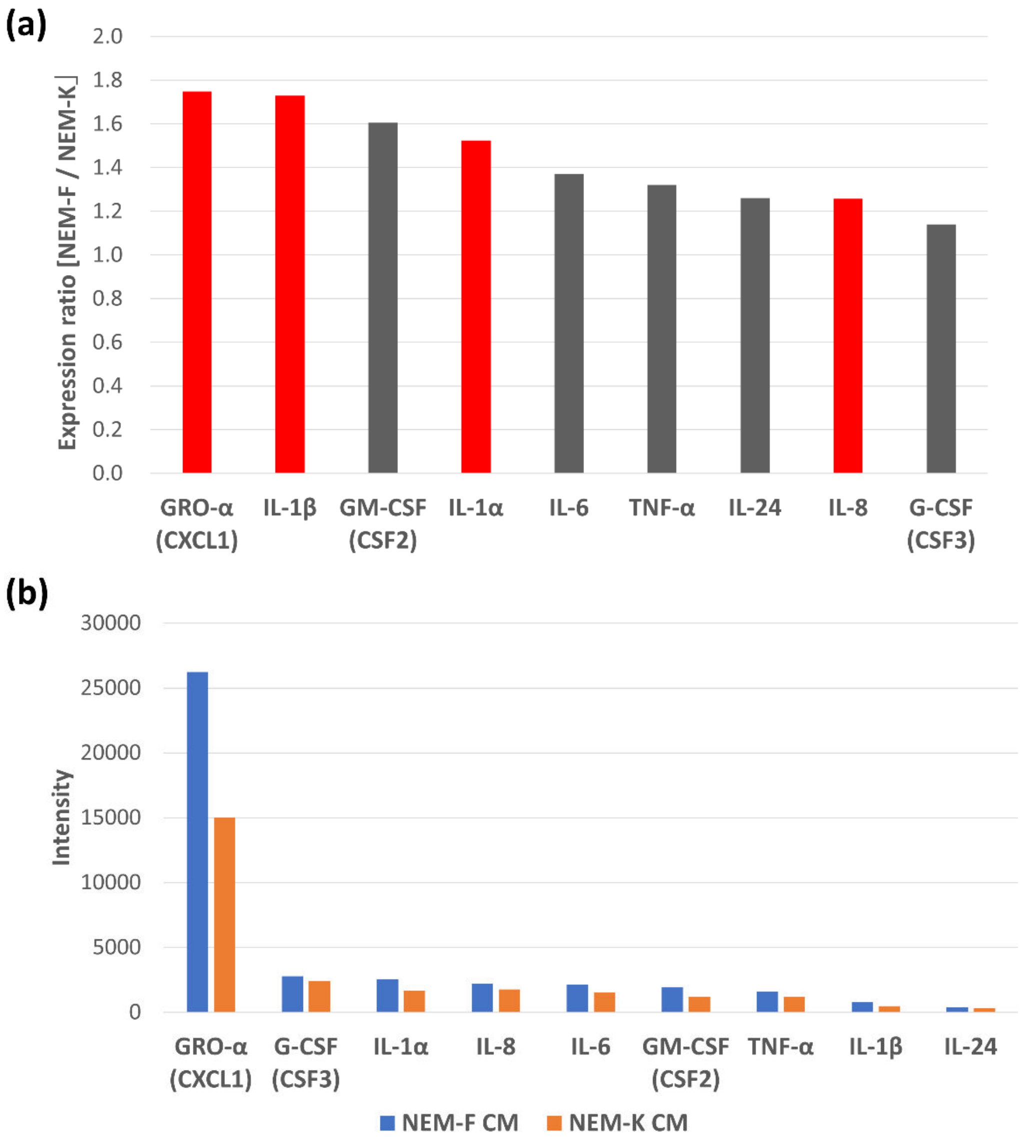

Figure S2. Results of protein array analysis between culture supernatants of NEM-F and NEM-K. Red bars represent the selected inflammatory cytokines based on the results of gene expression microarray analysis. Expression ratio (A) and intensity (B) in NEM-F relative to that in NEM-K.

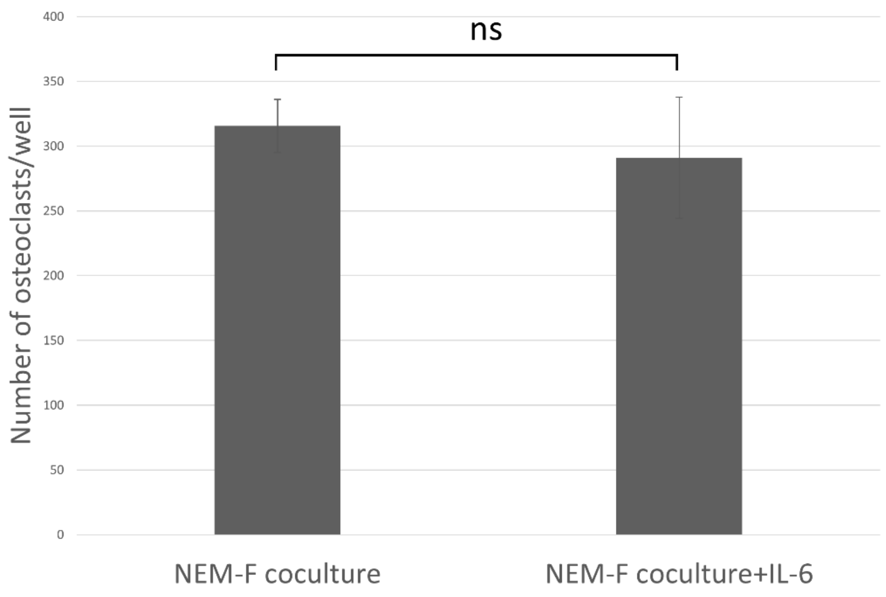

Figure S3. Quantitative data on the number of osteoclasts induced by the coculture of NEM-F cells with OPCs with or without supplementation of IL-6. Ns: not significant (Student's *t*-test).

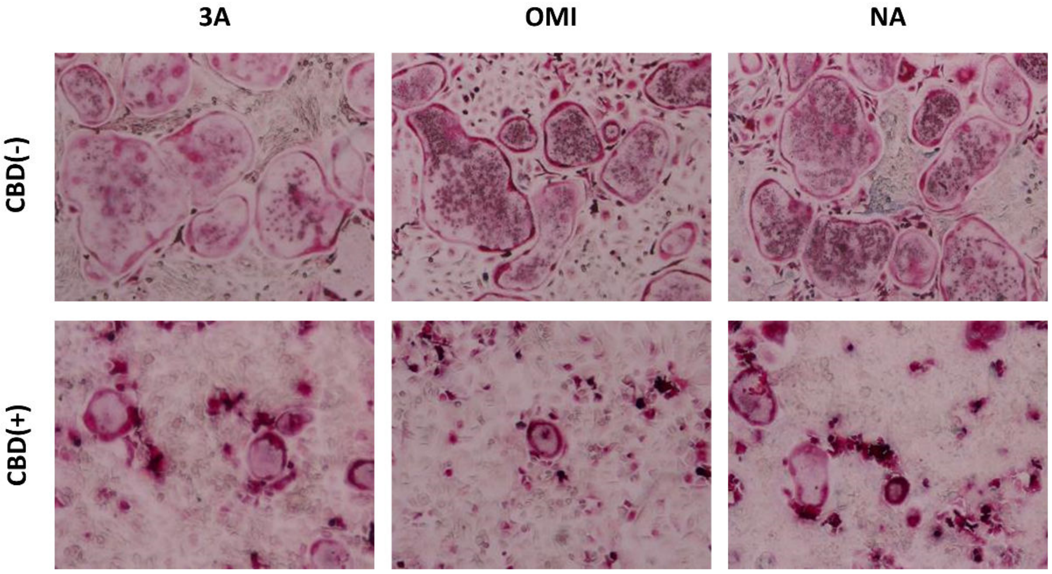

Figure S4. Representative views of TRAP staining 5 days after the coculture of OSCC cell line, 3A, OMI, or NA with OPCs with or without supplementation of CBD.
